# Public–Private Partnerships in Cloud-Computing Services in the Context of Genomic Research

**DOI:** 10.3389/fmed.2017.00003

**Published:** 2017-01-20

**Authors:** Palmira Granados Moreno, Yann Joly, Bartha Maria Knoppers

**Affiliations:** ^1^Centre of Genomics and Policy, McGill University, Montreal, QC, Canada

**Keywords:** public–private partnerships, cloud computing, genomic research, large-scale projects, MSSNG, ICGC, 1000 Genomes Project

## Abstract

Public–private partnerships (PPPs) have been increasingly used to spur and facilitate innovation in a number of fields. In healthcare, the purpose of using a PPP is commonly to develop and/or provide vaccines and drugs against communicable diseases, mainly in developing or underdeveloped countries. With the advancement of technology and of the area of genomics, these partnerships also focus on large-scale genomic research projects that aim to advance the understanding of diseases that have a genetic component and to develop personalized treatments. This new focus has created new forms of PPPs that involve information technology companies, which provide computing infrastructure and services to store, analyze, and share the massive amounts of data genomic-related projects produce. In this article, we explore models of PPPs proposed to handle, protect, and share the genomic data collected and to further develop genomic-based medical products. We also identify the reasons that make these models suitable and the challenges they have yet to overcome. To achieve this, we describe the details and complexities of MSSNG, International Cancer Genome Consortium, and 100,000 Genomes Project, the three PPPs that focus on large-scale genomic research to better understand the genetic components of autism, cancer, rare diseases, and infectious diseases with the intention to find appropriate treatments. Organized as PPP and employing cloud-computing services, the three projects have advanced quickly and are likely to be important sources of research and development for future personalized medicine. However, there still are unresolved matters relating to conflicts of interest, commercialization, and data control. Learning from the challenges encountered by past PPPs allowed us to establish that developing guidelines to adequately manage personal health information stored in clouds and ensuring the protection of data integrity and privacy would be critical steps in the development of future PPPs.

## Introduction

Public–private partnerships (PPPs) have been increasingly used to spur and facilitate innovation in a number of fields. Healthcare is one of them. Some examples include the PATH Malaria Vaccine Initiative, the Drugs for Neglected Diseases Initiative, the TB Alliance, and the International AIDS Vaccine Initiative. These partnerships usually involve a health authority, public hospitals or research centers, and private companies, usually pharmaceuticals. The purpose is commonly to develop and/or provide vaccines and drugs against communicable diseases, mainly in developing or underdeveloped countries (1). With the advancement of technology and of the area of genomics, these partnerships started focusing on genomic research projects that aim to advance the understanding of diseases that have a genetic component and to develop personalized treatments. This new focus has brought about other forms of PPPs. These new forms of PPPs involve information technology (IT) companies, which provide computing infrastructure and services to store, analyze, and share the massive amounts of data genomic-related projects produce.

This article explores models of PPPs proposed to handle, protect, and share the genomic data collected and to further develop genomic-based medical products. It also identifies the reasons that make the model suitable for these purposes and the challenges it has yet to overcome. To achieve this, the article describes the details and complexities of MSSNG, International Cancer Genome Consortium (ICGC), and 100,000 Genomes Project (100,000 GP), the three PPPs that focus on large-scale genomic research to better understand the genetic components of autism, cancer, rare diseases, and infectious diseases with the intention to find appropriate treatments. All the three projects require cloud-computing services (CCSs) to store, process, and share the massive amounts of genomic and health data that they collect. Both MSSNG and ICGC partnered with a commercial IT company that provides public CCSs. The 100,000 GP has a private cloud and partnered with private companies that do the sequencing of the DNA samples and that will develop diagnostic tools and treatments.

Our analysis compares these three genomic research projects, two of which (MSSNG and ICGC) include a well-established private IT company for the provision of CCSs as partners. We identify core elements about their organization, their goals, and current status and progress. The 100,000 GP has in-house (private) CCSs and partnered with private companies for other purposes [sequencing and interpretation assistance and translational research and development (R&D)]. This variation allows us to determine whether there is a difference between having a private specialized company handling the storage and sharing of genomic data or having in-house CCSs.

The sources we used for the description and analysis of the projects were the projects’ websites, documentation, and press releases without including internal private agreements with the companies that were not made public. Relying only on public documents, however, prevents us from learning about undisclosed challenges that the projects may encounter, their approaches to solve them, as well as private negotiations between the public and the private parties, all of which may limit our analysis. The sources we used for the context were peer-reviewed articles obtained from Google scholar using key terms such as “cloud-computing” and “public-private partnerships” alone and “cloud-computing” or “public-private partnerships” AND “health research,” “genomic research” or “medical genetics.” We also consulted Canadian, American, and British governmental websites and policies.

The article is organized into three sections. The first section presents the basic concepts of PPPs in the area of healthcare. This section intends to provide a general context on the characteristics, uses, stakeholders, benefits, and challenges of PPPs. The second section focuses on the three large-scale genomic research projects that use a model of PPPs and CCSs to reach their goals. It starts with basic concepts of cloud computing including characteristics, models of service, deployment models, and stakeholders and uses in healthcare. It then focuses on each of the three genomic research projects. For each project, we describe the purpose, current status, parties involved and roles, data location, conditions of elasticity in terms of costs and space, extent of control that the leading organizations of the project have over the data stored, access conditions, and confidentiality and privacy mechanisms. The third section discusses the lessons learned from all the three projects including the benefits that the projects have found in using a PPP model as well as the challenges that they still face.

## Context

### PPPs in Health-Related and Genomic Research

In medical research (e.g., genomic, pharmaceutical, clinical, etc.), the development of innovative results and products requires a constant targeting of new diseases or conditions to better understand the causes or factors associated with the disease, find and optimize new drugs and treatments, and address current safety, efficacy, and validation concerns. This challenging, complex, and constantly changing environment has encouraged parties from the public and the private sectors to intensify their collaboration (2). One common model of collaboration is that of a PPP with technological features that involves the use of CCSs.

A PPP is an agreement between the public and the private sectors to collaborate with each other by sharing objectives, resources, risks, and responsibilities (3). The partnership can be national or international, depending on its partners. The purpose of a PPP in the context of innovation is to jointly tackle the difficulties of the different stages of the innovation and translation processes and to make those processes more efficient. This purpose is based on the acknowledgment that the innovation and translation processes are too diverse, burdensome, and lengthy for either to take on alone (2–4).

Public–private partnerships have become a very important and prolific model to spur innovation, strengthen businesses, and more efficiently build and maintain public infrastructure. For instance, they have been used in projects to build and maintain roads, bridges, tunnels, railroads, airports, schools, and hospitals. They have also been used to build and operate prisons and to provide the infrastructure to solve water supply problems (3–6). Research in many fields, including health, has also used PPPs (7). Particularly in the context of healthcare and health-related research, many PPPs are created to make the process of R&D of drugs, vaccines, diagnostic tests, and medical devices more efficient and effective, as well as to improve patients’ access to medical innovative products (2, 4, 8, 9).

Different types of partnering may take place. Some include strategic innovation partnerships, consortia, joint research, crowdsourcing, outsourcing, licensing, incubator, and venture capital investments. The most common partners in PPPs in health-related research include public and private hospitals; public and private universities; non-for-profit organizations; patient organizations; small-, medium-, and big-size pharmaceutical and biotechnology companies; and governmental agencies. Technological advances in IT (e.g., the power of hardware, software, and mobility of devices), biomedicine, the different types of *omics* (e.g., genomics, proteomics, metabolimics, etc.), and the production of big data^1^ have attracted a new essential partner: IT companies (10).

Public–private partnerships are useful to improve access to health-care services as well as to spur R&D in the field (3). Since multiple partners will be able to combine their resources, infrastructure, and responsibilities, they may have the possibility to allocate their resources more efficiently and thereby use some of these resources to provide health-care services in a larger area. Furthermore, by combining resources, infrastructure, and skills from members of the public sector, PPPs may also help to focus on a specific condition that is endemic to a particular region where, because of the poor socio-economic conditions of its population, there is not a very profitable market and therefore potentially no interest from pharmaceutical companies. This focus may help to overcome situations of market failure and address diseases that would otherwise be neglected (3, 13). With respect to R&D, these partnerships could satisfy different needs, such as enabling access to new ideas or providing the perspectives of a broader community, overcoming complicated or extensive challenges such as regulatory approvals, transferring of knowledge or expertise, and establishing an earlier involvement with entrepreneurs (2).

Public–private partnerships allow the different partners to share resources that include economic funds, knowledge, expertise, information (e.g., data and samples and patient base), and infrastructure. They also enable partners to share risks, responsibilities, and networks of experts while accessing new ideas from a broader community. Sharing economic resources, risks, and responsibilities can alleviate the burdens inherent in the innovation process making it less costly and faster. For instance, it can avoid or decrease the need for costly initial capital investments. Sharing expertise, ideas, information, and infrastructure can make the process more efficient, more fluid, and more interconnected. Overall, these benefits increase the value of the investment and infrastructure of all the partners as well as the value of the project the PPP was set for (2–4).

Public–private partnerships can also democratize the innovation process, as more agents, and not only those with all the initial necessary resources, can participate. For instance, small- and medium-size companies could enter a PPP and participate in large-scale projects, which they would not be able to do, if they depended entirely on their own means and resources. This democratization could also benefit developing countries, as the governments and companies of these countries could be able to participate in projects that, because of the costly resources that they require, are more technologically and scientifically advanced and usually take place in developed countries. This could ultimately promote the economic, scientific, and technological growth of these countries, as employment would be created, distribution of goods would be increased, and technological and scientific advancements would be transferred (14).

However, despite the abovementioned advantages of PPPs, there are certain difficulties that need to be considered and overcome. First, the parties entering the partnership may have unrealistic expectations about what the partnership is supposed to create. Second, the parties from the public sector tend to have objectives and interests that differ from those of the parties of the private sector. Third, the negotiation process to achieve these partnerships is complicated. This is even more serious in partnerships whose projects are caught in the middle of political debates or public opposition because of their focus or the subject matter of those projects. Fourth, there may be inadequate legal or regulatory frameworks, which could make the partnership more difficult to set or to maintain. Fifth, these partnerships need to be maintained, particularly in health-related research, as the processes of R&D in this field tend to be lengthy. These difficulties make the coordination and management of partnerships challenging (2–4, 7).

In order to address and even overcome some of the abovementioned difficulties, certain reforms have been suggested. For instance, it has been proposed that those who have the authority to make decisions in the project be involved in the R&D process from its inception. This would allow them to act in a timely manner. Additionally, the coordination and decision-making process needs to be mindful of the specific field(s) associated with the project. Progress and deliverables need to be continuously monitored in order to increase the chances of detecting any hurdle or setback that may arise. Work methods and agreements should be clarified. It is also recommended that political, legal, commercial, operational, environmental, and economic risks and responsibilities be distributed among all the partners in a timely fashion. For this, they should be identified and distributed among the partners in accordance with the partners’ financial and technical capabilities for management. In addition to all these recommendations, maintaining mutual confidence and trustworthiness throughout the project is one of the most important suggestions to ensure that the PPP succeeds (3, 7, 15).

### Cloud Computing in Genomic Research

In health-related research, and particularly regarding genomic research, the involvement of IT companies in PPPs is essential. The development of devices to continuously collect data from patients created massive amounts of data. Furthermore, the advent of genomics and other *omics* fields and of technologies that accelerate the collection of data have also contributed to the production of massive, complex, and potentially helpful data (10, 12, 16). These data need to be stored, organized, analyzed, and made accessible to researchers and developers in order for them to discern patterns, associations, and trends for the better understanding of diseases in order to create improved and customized diagnostic tests, drugs, vaccines, and treatments (10, 11). These needs often create problems of interoperability among the different sources of the aforesaid data and platforms, high costs of infrastructures, and other necessary technological tools. Hence, trained personnel are required to manage, operate, and maintain the infrastructure as well as to be capable to analyze, process, and share the data (12, 17). In order to address these problems, CCS providers are desirable partners in genomic research partnerships (14–18).

Cloud computing is defined “as a model for enabling ubiquitous, convenient, on-demand network access to a shared pool of configurable computing resources (e.g., networks, servers, storage, applications, and services) that can be rapidly provisioned and released with minimal management effort or service provider interaction” (19, 20). Based on this definition of the National Institute of Standards and Technology, CCSs are provided on demand (server time or storage), accessible through the use of standard mechanisms over a broad network, immersed in a resource pool serving multiple consumers, and elastic. The elasticity allows multi-tenancy (i.e., one single application can serve multiple users) (16, 20, 21).

Cloud-computing services can be deployed in a private cloud, a community cloud, a public (commercial) cloud, or in a hybrid cloud. A private cloud provides exclusive services to one single organization, and the infrastructure (data center) is owned, managed, and operated either by that same organization that uses it or by a third party. A community cloud provides services to a specific group with a common interest, and the infrastructure may be owned, managed, and operated by one or more organizations in the group or a third party. A public cloud provides services to the general public, and the infrastructure is owned, managed, and operated by a company, an academic or governmental organization, or a combination of both. A hybrid cloud is composed of two or more clouds that can be private, public, or community (15, 16, 18, 20, 22).

Cloud-computing services are offered in three models: infrastructure-as-a-service,^2^ platform-as-a-service,^3^ and software-as-a-service^4^ (15, 16, 20, 22). Some authors also include mobile backend-as-a-service^5^ (21, 23). Four types of stakeholders are associated with CCSs in health-care fields. The first ones are end-users (also called consumers). They use the services on demand based on their own specific needs. End-users include physicians, medical staff, patients, medical researchers, and IT experts. Patients use CCSs for purposes of personal health-care management. Medical researchers and staff use them for medical and genomic research. Finally, IT staff employs CCSs for the creation and implementation of cloud-based solutions (16). The second type of stakeholders refers to service providers (companies and organizations). They own, operate, maintain, and update the system and provide the service to the end-users. The enablers are the third type of stakeholders. Even though not essential, they sell products and services to facilitate the delivery and use of the CCSs. Finally, the regulators are in charge of providing guidelines, standards, and rules regarding the proper and ethical use of CCSs. These regulators can be national or international. Regulations can focus on privacy, security, liability, intellectual property, cross-border issues, access and transfer, governmental extent of monitoring and access, and forensics (21).

Cloud-computing services bring a number of benefits. For example, they allow companies and organizations to use computing resources (e.g., storage and analytical computing) as a utility on a pay-per-use basis. This condition results in costs reduction, as the infrastructure needed is “rented,” rather than having to invest in building and maintaining the infrastructure. These reduced costs allow any company or organization, including those that are small and medium sizes, to enter the research field, potentially also benefiting companies, organizations, and entities in developing countries. The latter could avoid costly investments while using and exploiting state-of-the-art computing processes. CCSs can rapidly and easily scale-up or scale-down on a need-to basis. This situation gives customers flexibility to add, expand, reduce, eliminate, or re-distribute their resources and services. Moreover, companies and organizations can hire on-demand self-services and decide the location of the server where their data will be stored. IT experts are responsible for maintaining the infrastructure, thus making the upkeep of the cloud easier. Customers can share resources and costs among themselves, allowing a more efficient use of resources and reduced costs and timelines. The use of CCSs can also increase productivity in the development of the projects undertaken by customers, given that multiple users can work simultaneously on the same data and collaborate without the need for constant software upgrades. Along with this efficient use of resources, CCSs also reduce the time that clinical and genomic research takes. Usually, these processes require researchers to download or upload data to their local computers in order to process, analyze, and obtain results. These results then need to be uploaded to repositories for publishing and sharing. Since cloud computing eliminates the need to download the data, the whole process is shortened. In addition, given that there is no need to build and maintain individual infrastructure each time a project requires computing services, the use of CCSs could be considered more environmentally friendly. Finally, even though there are still concerns about losing control over certain data, the centralization of the data, the increased security-focused resources (e.g., encryption tools and techniques, firewalls, auditing capabilities, etc.), and the specialization of IT personnel that focuses on these matters are ways to increase the level of security (15, 21, 22, 24).

The most serious challenge with CCSs in healthcare is the perceived risk of possible loss of control of health data, jeopardizing the safety and security of data and patients’ privacy and confidentiality. A proposed solution is the use of controlled access, audit control, authentication, authorization mechanisms, as well as transmission and storage security. Some of these tools include secure transmission protocols, special security certificates, access control lists, electronic keys, and digital signatures. However, these measures may be inconvenient, particularly in emergency situations. The use of tools that allow the anonymity of data has also been suggested,^6^ but anonymization prevents the return of health results. Data integrity may also be at stake due to hacker attacks, network failures, or poor management. Again, the use of controlled access, authentication, authorization, and transmission and storage security measures can also help to preserve data integrity. Ensuring that CCS providers have appropriate backups and retrieval measures in place can be also helpful. Another concern regarding the use of CCSs is reliability. Cloud-computing providers can suffer outages (e.g., Amazon’s outages in 2008, 2011, and 2012). They may also suspend certain services either temporarily or permanently (e.g., Google Health). CCS providers also have to ensure compliance with data privacy laws of the specific locations of the data centers where data are stored and transferred. Getting end-users to trust CCSs and providers, particularly with respect to data security and privacy, is perhaps the greatest challenge (15, 16, 22, 24).

Irrespective of these challenges, CCSs have been used in several areas of healthcare: telemedicine/teleconsultation,^7^ medical imaging,^8^ public health and patient self-management,^9^ hospital management and information systems,^10^ therapy,^11^ and secondary use of data (16). Genomic research in particular benefits from the secondary use of data. Since CCSs enable the storage of massive amounts of data and the use of complex computing processes at reduced costs, it is possible to simultaneously do clinical and genomic data analysis, text mining, and ongoing clinical research (16).

By enabling global access to clinical and genomic data and other resources, CCS providers unlock the value of big data, and particularly of genomic data. Global access to such data allows scientists, researchers, and developers to use data generated in different regions, from different patients, and with different techniques. It also allows researchers and developers from different backgrounds and multiple disciplines to work together simultaneously. This diversity enables them to better identify and understand patterns, variants, and correlations among the multitude of factors that cause or prompt a disease so as to innovate and eventually provide accessible health-care services (14, 15). These advantages constitute the rationale for the involvement and uptake by research centers, academic institutions, and governmental agencies in PPPs. Private companies have reasons as well for getting involved in CCSs and in PPPs in the area of genomic and medical research. The first reason is economic. Given the advantages posed to end-users, the demand for these services has greatly increased in the past and is expected to grow even more within the next few years. In 2014, the value of the cloud-computing industry was expected to be $150 billion. Furthermore, companies providing public, community, and hybrid clouds are able to avoid the underutilization of their infrastructure and of their other resources. The third reason is that some companies are increasingly interested in contributing to improving the population’s health (21).

Governmental policies and regulatory frameworks can help to create favorable conditions for the success of PPPs and the use of CCSs (3). The “Cloud First” policy in the U.S. is an example of a governmental policy that supports and encourages partnerships with new technology.^12^ Laws and policies that address issues of consent, privacy, and personal data applicable in the context of cloud computing can also facilitate the use of CCSs, as they can increase the trust of the general public, research institutions, and funding agencies on projects involving cloud-computing technology (3, 15, 27).

For example, in general terms, consent rules usually require that any use or disclosure of health information is previously authorized in writing by the individual to whom the information belongs. However, recent consent policies and projects have changed so as to adapt to and benefit from recent practices of democratization of access of data, by which many researchers from different centers share and analyze data. MSSNG is an example (14, 28, 29).

In the U.S., the Health Insurance Portability and Accountability Act (HIPAA) provides national standards for the protection of certain health/medical information (privacy rule) and for the protection of this information when it is held or transferred in electronic form (security rule). The main purpose of HIPAA is to allow covered entities^13^ to adopt new technologies in order to improve patients’ health while still protecting the privacy of individuals’ health information. The health information protected is “all individually identifiable health information^14^ held or transmitted by a covered entity or its business associate,^15^ in any form or media, whether electronic, paper or oral.” CCS providers can be considered business associates. HIPAA can encourage the use of CCSs, as it allows the donors of genomic data to trust that risks to the safety of their data or to their privacy will be minimized^16^ (15, 22, 28, 29). In addition to HIPAA, the U.S. has the NIH security best practices for controlled access data. This document states the minimum standards of National Institutes of Health (NIH) regarding the management and protection of controlled access to data transferred to and maintained by institutions either in their own data centers or in CCSs. The use of encryption and firewalls, as well as access control procedures, is suggested as protection mechanisms (14, 30).

Canada’s Personal Information Protection and Electronic Documents Act (PIPEDA) regulates the way in which members of the private sector collect, use, and disclose personal information^17^ in commercial activities. This includes information collected and/or stored online. According to PIPEDA, organizations or companies are only allowed to use or disclose the information they collect on the terms agreed at the time of collection, unless a new expressed consent of the individual who provided that information is obtained (22, 31–33). Similar to HIPAA, PIPEDA and the NIH’s best practices can encourage the use of CCSs by obligating CCSs providers to adopt measures that protect the safety of health data and the donors’ privacy.

## Selected Projects

The advancement of DNA sequencing techniques has facilitated and accelerated the generation of genomic data. The costs of sequencing have also decreased. These new conditions have prompted the launch of large-scale projects that involve members of the public and private sectors playing different roles (14). Some examples of PPPs that involve CCSs for genomic research include (1) the Collaborative Cancer Cloud that partners Intel, the Knight Cancer Institute at Oregon Health and Science University, the Dana-Faber Cancer Institute, and the Ontario Institute for Cancer Research; (2) the Cancer Genome Collaboratory (CGC), whose partners are the Open Cloud Consortium, the Ontario Institute for Cancer Research, and the Bionimbus Protected Data Cloud; (3) MSSNG that partners Autism Speaks and Google; (4) 100,000 GP which joins Genomics England (GE) with AbbVie, Alexion Pharmaceuticals, AstraZeneca, Berg Health, Biogen, Dimension Therapeutics, GCK, Helomics, NGM Biopharmaceuticals, Roche, and Takeda; and (5) the ICGC that partnered with Amazon Web Services (AWS). We focus on the last three (Table 1).^18^

**Table 1:** **Summary of the selected cases**.

	MSSNG	International Cancer Genome Consortium (ICGC)	100,000 Genomes Project
Launch date	2014	2008	2012

Focus	Autism	Cancer	Rare disorders, infectious diseases, and common cancers

Goals	To collect and sequence DNA from 10,000 families to understand causes of the autism condition across its wide spectrum, identify its different subtypes, and help in the development of more personalized and accurate treatments	To collect and sequence 50,000 genomes. To coordinate and generate current and future large-scale cancer genome studies in tumors from 50 different types and subtypes of cancer in order to identify common patterns of mutation and understand the biology of cancer	To collect and sequence 100,000 genomes from 75,000 patients. To enable new scientific discovery and medical insights regarding the selected rare disorders, infectious diseases, and common cancers for the creation of better tests, better drugs, more accurate and timely diagnoses and treatments, and more personalized care

Public and private partners	Public sector: Autism Speaks-Autism Genetic Resource Exchange and Toronto’s Hospital for Sick Children	Public sector: 25 research centers from around the world, the TCGC, and Cancer Genome Collaboratory (CGC)	Public sector: Genomics England (GE), National Institute for Health Research, Public Health England, Health Education England, 13 National Health Services (NHS) Genomic Medicine Centres, GE Clinical Interpretation Partnership (GeCIP) partnership, and 85 NHS trusts and hospitals
	Private sector: Google	Private sector: Amazon Web Services (AWS)	Private sector: Illumina, Congenica, WuXi NextCODE, Omicia, Cypher Genomics, Nanthealth, Genomics Expert Network for Enterprises (GENE) Consortium, and GeCIP partnership

CCS provider/type	Google—private company offering public cloud-computing services (CCSs)	AWS (Amazon)—private company offering public CCSs and CGC—public academic resource offering private CCSs	GE—company owned by the U.K. Department of Health offering private CCSs

Status/progress	7,000 whole genomes sequenced. The project has provided access to more than 100 investigators from 9 countries	10,000 whole genomes sequenced and 89 cancer genome projects. ICGC Data Access Compliance Office (DACO) has approved access to 734 projects (including renewals) over the years	16,171 whole genomes sequenced. No mention of projects/researchers approved to access to the data

Characteristics of services	Scalable, elastic, on-demand, and accessible through a broad network. Specialized service for big genomic data	On-demand, elastic, and scalable. Specialized for massive data	Scalable and elastic. Members are encouraged to nominate new diseases

Security and safety	Encryption, online backup, and disaster recovery. ISO 27001, SSAE-16, SOC1, SOC2, and SOC3 certifications	Encryption. ISO 9001, ISO 27018, SOC1, SOC2, SOC3, FISMA, PCI DSS, ITAR. FIPS 140-2 certifications. The AWS Data Processing Agreement is approved by Art. 29 Working Group. Access approved by ICGC DACO	Encryption. BS7799 and ISO27001 certifications. No raw data can be downloaded or withdrawn. Access Review Committee assesses access requests. GENE members pay a fee for access

Benefits of the specific public–private partnership for CCSs	Access to Google’s massive infrastructure, capabilities, geographical presence, and experience handling sensitive and personal data. Compliance with the laws of several jurisdictions. No special application or program needed. Enabling of global and multidisciplinary collaboration and accelerated research and development (R&D). No initial investment for cloud-computing capability needed. Potential trust on the project because of Google’s involvement. Diverse and enriched analysis due to possibility to download data.	Access to AWS’ massive infrastructure, capabilities, geographical presence, and experience handling sensitive and personal data. Compliance with the laws of several jurisdictions. No special application or program needed. Enabling of global and multidisciplinary collaboration and accelerated R&D. No initial investment for cloud-computing capability needed. Potential trust on the project because of Amazon’s involvement. Diverse and enriched analysis due to possibility to download data.	Private companies’ involvement in the data’s sequencing and the translational process is expected to accelerate the R&D process. Specially tailored for the project’s needs, circumstances, and UK regulations. GE maintains exclusive control over the stored data. Data cannot be downloaded, and they never leave the data center, which grants GE higher control over the data. Given GE’s nature (owned by U.K. Department of Health as opposed to being owned by a private company), participants may trust the project more.

Challenges	Potential distrust on the project because of Google’s involvement. Even MSSNG having control over the access to the stored data, Google is in charge of the data’s security, safety, and integrity. Less control over the data, as they can be downloaded. Confidentiality and privacy are still unresolved: re-identification of participants is possible. Reconciliation of different expectations and goals of public/private sectors.	Potential distrust on the project because of AWS’ involvement. Even ICGC having control over the access to the stored data, AWS is in charge of the data’s security, safety, and integrity. Less control over the data, as they can be downloaded. Confidentiality and privacy are still unresolved: re-identification of participants is possible. Reconciliation of different expectations and goals of public/private sectors.	Geographical expansion requires ensuring compliance with new jurisdictions. Large investment needed to start and maintain the CCS. Reduced GE’s experience handling massive amounts of data in comparison with Google’s or AWS’ and potential less trust on GE’s capacities to secure the data. Open access/open science vs. no download policy. Confidentiality and privacy are still unresolved: re-identification of participants is possible. Reconciliation of different expectations and goals of public/private sectors.

### MSSNG

The project, which was launched in December 2014, is sequencing and analyzing the DNA of 10,000 families affected by autism. The aim of this project is to help to understand the causes of the condition across its wide spectrum, identify its different subtypes, and help in the development of more personalized and accurate treatments. It seeks to promote and enable open science research and lead to a better understanding of autism. The name of the project has the vowels of the word “missing” deliberately omitted symbolizing the missing pieces of the autism project. The project is a collaborative effort of Autism Speaks, Toronto’s Hospital for Sick Children (Centre for Applied Genomics), and Google. It also has funding contributions from KRG Children Charitable Foundation, the Canadian federal government and the province of Ontario, Gordon and Llura Gund Foundation, Mel Karmazin Foundation, and the Allerton Foundation. MSSNG’s key leaders are David Glazer (Google), Mat Pletcher (Autism Speaks), and Stephen Scherer (Toronto’s Hospital for Sick Children and University of Toronto) (34, 35).

In April 2016, the project released more than 5,000 whole genome sequences. In August 2016, this number rose to 7,000. MSSNG includes 3,000 genome sequences from participants in the Autism Genetic Resource Exchange (AGRE) (35, 36). Until now, MSSNG has granted access to its database to more than 100 investigators from 9 countries. Access to this database has enabled research projects on several aspects associated with autism. For instance, in August 2016, researchers from the Toronto’s Hospital for Sick Children published a study in the *npj Genomic Medicine* journal on the genome-wide characteristics of the novo mutation in autism and on the epigenetic risk factors for autism^19^ (38).

MSSNG has several partners. Autism Speaks is a non-for-profit organization and advocacy group located in New York, U.S. Its involvement in MSSNG is associated with the awareness, fund raising, and support of basic, translational, and clinical research and projects such as AGRE. AGRE is a DNA repository and family registry of genotypic and phenotypic information funded by Autism Speaks. The Toronto’s Sick Children Hospital is a public hospital located in Toronto, ON, Canada, with governmental funding specializing in children and affiliated to the University of Toronto. Its involvement in the project is on the scientific research area. Both parties—Autism Speaks-AGRE and Toronto’s Sick Children’s Hospital—represent the public sector and provide the genomic data, health information, and the scientific/medical analysis.

Google represents the private sector in MSSNG. The partnership with Google outsources CCSs to store, process, and share the genomic data collected by Autism Speaks and Toronto’s Sick Children’s Hospital with researchers around the world. Google is a multinational company focused on Internet and computer-related services and products whose objective is to “organize the world’s information and make it universally accessible and useful” (39). As such, it has created tools that help to organize almost all kind of information and fulfill its objective. For instance, Google’s Search, Maps, and YouTube allow users to access documents, images, information, videos, and roads and make that information useful to satisfy their individual needs. Google Cloud Platform supports the aforesaid services and helps users to do something similar with their own information. It also helps to store, organize, analyze, process, and make accessible complete medical/health records, including genomic data. Google Genomics was conceived to specialize on big genomic data (12, 14). Furthermore, Google has long-supported the open source philosophy and has implemented philanthropic programs in areas such as climate change, public health, and poverty. In view of this, it would be safe to assert that one of the reasons for which Google entered this PPP with Autism Speaks and Toronto’s Sick Children Hospital could be its genuine interest in maintaining its involvement in the efficient management of big data and contributing to a deep understanding of autism and the development of personalized medicine that will help to manage this condition (14, 40–42).

The MSSNG database contains research data,^20^ whole genome sequencing (WGS) data, and researchers provided data (43). This information is stored, processed, and made available through Google’s infrastructure. First, the data are uploaded to Cloud Storage and then imported to Google Genomics. The latter allows researchers to access the data using Genomics APIs, explore it with Google BigQuery, and analyze it with Google Compute Engine (44).

Google’s CCSs are scalable, elastic, provided on-demand, and accessible through a broad network. They offer predefined or custom machines with persistent disks or local SSD,^21^ support for Linux and Windows, and encryption for the data on the fly before being transmitted. These conditions allow MSSNG to scale-up or scale-down as needed. Furthermore, Google’s storage service is highly durable with online backup and disaster recovery (45, 46). This, along with Google’s long-term experience in IT, makes it the most suitable party in the PPP to be responsible for the CCSs of the project and for the integrity of the data. Moreover, the project’s nature makes its success dependent on the existence of a large network of researchers that contribute to the advancement in the understanding of autism and on the reliability and stability of the cloud-computing infrastructure and services. Given Google’s global and powerful infrastructure, partnering with it to obtain cloud computing can enable the large global network of researchers that the project needs and can support a long-term project.

In terms of security and safety, MSSNG also seems to maintain a reasonable level of control over the data stored. Google’s Data Processing and Security Terms state that while Google processes the data submitted by the customer (in this case, Autism Speaks and Toronto’s Sick Children Hospital), it is the latter who controls the data. Google’s processing is only performed in accordance with the customer’s instructions (46, 47). Access is provided by Data Access Compliance Office (DACO) to anyone complying with the project’s access and privacy policies, which include the use of encrypting tools (48, 49). To ensure this, Google has “technical and organizational measures to protect the data against accidental or unlawful destruction or accidental loss or alteration, or unauthorized disclosure or access” in place, including several layers of encryption to protect the data. This includes ensuring that its staff complies with these measures. Google will promptly notify the customer of any security breach and will immediately take the reasonable steps to minimize the harm. Google’s security and privacy measures comply with the ISO 27001, SSAE-16, SOC 1, SOC 2, and SOC 3 standards (46, 50). The project’s data will be stored where MSSNG chooses between the U.S. or Europe or another location setting offered by Google (46, 51). Either Autism Speaks and Toronto’s Sick Children Hospital or whoever collects the data is responsible for obtaining the necessary consent for the data to be stored and processed by Google (47). Finally, all changes to Google’s Data Processing and Security Terms or to the services will be posted online (47).

### ICGC and the Cancer Genome Atlas (TCGA)

The project was launched in 2008 with the intention of coordinating and generating current and future large-scale cancer genomes studies in tumors from 50 different types and subtypes of cancer (52, 53). For this, genomes of at least 50 types of cancer will be collected, mapped, and shared. The objective of coordinating and generating numerous large-scale cancer genomes studies rises from the potential to identify common patterns of mutation and understand the biology of cancer found in using systematic genome-wide screens (54).

The headquarters of the project are located in the Ontario Institute of Cancer Research in Toronto, ON, Canada. The list of current ICGC members include over 25 research centers from Australia, Canada, China, Europe, France, Germany, India, Japan, Mexico, Singapore, Saudi Arabia, South Korea, Spain, the U.K., and U.S.^22^ All entities are allowed to become members, provided that they comply with the ICGC’s principles and guidelines (55). There are different types of scientific partnerships/collaborations. ICGC funding members of large-scale projects are expected to have approximately 500 patients per sample and an estimated contribution of USD $20 million. Those who contribute with less than 500 samples (at least 100) and less than USD $10 million are members with affiliate status. Research members are centers that acquire and analyze samples for one or more cancer genomes and that are nominated as such by a funding member, who will provide the necessary funds for them (52, 55).

TCGA is a collaboration between the National Cancer Institute and the Human Genome Research Institute to create comprehensive and multidimensional maps of genomic changes in 33 types of cancer. TCGA is one of the world’s largest collections of cancer genome data available from more than 11,000 donors. TCGA created a pipeline to collect, select, and analyze human tissues on a very large scale. Its funding is federal from the National Cancer Institute, the NIH, and the Department of Health and Human Services. The project is scheduled to end in 2017 (56, 57).

There are currently 89 cancer genome projects examining different types of tumors. The tumors being examined are from the biliary tract, bladder, blood, bone, brain, breast, cervix, colon, eye, head and neck, kidney, liver, lung, nasopharynx, oral cavity, ovary, pancreas, prostate, rectum, skin, soft tissues, stomach, thyroid, and uterus (55). The datasets of TCGA are also stored and released through AWS. The ICGC and TCGA have collected, stored, analyzed, and released data from 10,000 genomes. By 2018, the ICGC project intends to have over 50,000 genomes (15, 53, 56).

In addition to the scientific partners mentioned above, the project has academic and commercial partners that provide CCSs. The CGC is the academic partner, and AWS is the commercial partner in this hybrid cloud. The CGC was built and is led by the Ontario Institute for Cancer Research. This cloud stores data that are restricted to a small group of beta testers approved by ICGC DACO. The data stored in the CGC are provided by 25 projects and 14 primary sites. There are two data centers: SciNet (Toronto) and Bionimbus (Chicago) (58, 59). AWS hosts data obtained from 20 projects and 12 primary sites. The datasets are hosted at the U.S. East (Northern Virginia) EC2 facility and are accessible in 190 countries (54, 58–60).

Amazon is one of the world’s largest providers of CCSs. The services operate from several geographical regions: U.S. East (Northern Virginia), U.S. West (Northern California and Oregon), Brazil (Sao Paulo), Europe (Ireland and Frankfort), South Asia (Mumbai), Southeast Asia (Singapore), East Asia (Tokyo, Seoul, and Beijing), Australia (Sydney), and GovCloud (Northwestern U.S.). Canada, China, India, Ohio, and the U.K. have been announced for 2017. All of the data and services stay in their designated region. Each region has multiple separated data centers to prevent outages from spreading. In December 2014, AWS was operating 1.4 million servers. The customer chooses the region in which its data will be stored. ICGC chose U.S. East (Northern Virginia) (61–63).

The CCSs provided by AWS are on-demand, low-cost, pay-as-you-go, and scalable. The services run in Mac, Linux, and Windows. Like Google, AWS has services specially tailored for scientific computing that adapt to the needs of massive datasets (60, 64, 65). AWS will notify its customers of any change to the services provided (63). ICGC is responsible for ensuring that the data comply with the respective applicable laws (66). Some of the AWS resources may be replaced or terminated. In these cases, AWS will not be held responsible for any damages, liabilities, or losses resulting from those replacements or terminations (66).

Amazon Web Services offers strong security measures and tools that have been certified and accredited, data encryption at rest, and in transit to manage the data. For instance, AWS security measures and tools have the SOC1, SOC2, SOC3, FISMA, PCI DSS, ISO 27018, ISO 9001, ITAR, and FIPS 140-2 certifications. Furthermore, the AWS Data Processing Agreement, approved by the Article 29 Working Group, meets the standard provisions of the European Commission with respect to data protection when data is transferred between clouds and regions. In accordance with the AWS privacy terms, AWS will only disclose the content stored in its cloud if it is mandated by law or a binding order of a governmental or regulatory body. AWS also offers reliable backup services. However, the customer, in this case ICGC, is responsible for the operation, maintenance, use, security, protection, and backup of the data. Finally, given the specialization on genomics/health-related data, AWS expressly states that they comply with HIPAA (60, 62–65, 67–71).

The ICGC member nations agree to core bioethics principles and elements for informed consent, privacy, and access. These requirements include that the data and samples will be used for cancer research, that they will be made available to the international research community through an open or controlled access database, and that this availability will be done under terms and conditions that will maximize the participant’s confidentiality (55). The data are made rapidly and freely available to the global research community through the ICGC data portal. Researchers have access to open and controlled portions of the data and to a number of user interfaces that address simple gene-oriented queries as well as those involving genomic, clinical, and functional information (15, 72).

The access to the ICGC data stored and processed in the AWS cloud is approved by ICGC DACO, which utilizes user authentication and authorization (e.g., decryption keys) to ensure safe access. Until today, 734 projects (including renewals) have been approved to have access to the ICGC data. Access to TCGA data is approved and done through the Cancer Genomics Cloud being Seven Bridges Genomics, the entity responsible for authorizing access to the data. This access and collaboration are expected to help researchers to identify commonalities and differences among different types of cancer. Other AWS customers/projects in the area of healthcare include NASA NEX, 1000 Genomes Project, TCGA, GenomeNext, and the Human Microbiome Project (56, 64, 67, 73, 74).

Amazon’s official motive for entering such a partnership is its interest in helping to “achieve major healthcare breakthroughs and unlock the mysteries of the human body” through the use of bioinformatics. This is also one of the main reasons for them to develop open source tools (73). The ICGC project definitely benefits from the AWS’ massive infrastructure, capabilities, geographical presence, and experience in safely and efficiently handling these amount of sensitive and personal data. It also benefits from the flexibility and adaptability of the services to the project’s specific needs. The cloud-computing partnerships with AWS and CGC allow scientists to access, search, and analyze ICGC without the need to install any special application or program or to download any of the datasets. This enhances collaboration and accelerates the R&D of tools and treatments for cancer patients (58, 60).

### 100,000 GP

The project, launched in late 2012, aims to “enable new scientific discovery and medical insights” and to “kickstart the development of a U.K. genomics industry.” It intends to create better tests, better drugs, more accurate and timely diagnoses and treatments, and more personalized care to save lives. The project focuses on rare disorders, infectious diseases, and common cancers. In order to achieve this, the project created 13 National Health Services (NHS) Genomic Medicine Centres to recruit approximately 75,000 patients to provide DNA samples and clinical information for the collection and analysis of 100,000 genomes between 2015 and 2017 (75–80).

In March of 2015, patients were already being diagnosed under the 100,000 GP. By December 20, 2016, the project had sequenced 16,171 genomes (81, 82).

Genomics England is in charge of the 100,000 GP. It is a company funded in 2013 by the Department of Health. It manages the contracts with U.K.-based companies, universities, and hospitals regarding the supply of services of sequencing, data linkage, and analysis. GE is also responsible for the secure storage of personal data as per NHS rules (75).

The project’s main scientific partners are the National Institute for Health Research, NHS England, Public Health England, Health Education England, and 85 NHS trusts and hospitals across England (75). While GE funds the WGS for patients of the NHS England Genomic Medicine Centres, the Northern Ireland Executive and the Medical Research Council funds the Northern Ireland Genomic Medicine Centre. Scotland and Wales have recently joined the project. The company Illumina is the private partner responsible for sequencing the DNA in the Wellcome Trust Genome Campus in Hinxton, U.K. Illumina will also develop a platform to improve and automate genome interpretation (77–79, 83). The project also partnered with other four companies to help with clinical interpretation: Congenica, WuXi NextCODE, Omicia, Cypher Genomics, and Nanthealth (79, 84).

In addition to these partnerships, the project has also partnered with the Genomics Expert Network for Enterprises (GENE) Consortium, composed of private companies, and the GE Clinical Interpretation Partnership (GeCIP), composed of researchers and clinicians from academia and the NHS researchers. There are currently 10 members in the GENE. Their goal is to accelerate the development of new diagnostics and treatments. On the other hand, members of the GeCIP help to interpret genomic data in a clinical context. Any individual, student, or staff member affiliated with a university or academic research institution, NHS trusts or authorities, charitable organization related to the 100,000 GP, U.K. and foreign governmental departments that carry significant research activity, or foreign health-care organizations (public or private) that undertake significant research activity can apply to become a member of GeCIP. Exceptionally, individuals affiliated with private U.K. health-care institutions, commercial companies, or self-employed can become members of GeCIP (85–88).

The raw data collected and stored by the 100,000 GPs are kept in GE’s data centers. In contrast to our two previous cases, no raw genome data can be downloaded and withdrawn from GE’s data center. Doctors, nurses, and health-care professionals in the NHS Genomic Medicine Centres have access to the data of their patients for health-care purposes. Researchers and companies need to apply to have access, but the computational analysis is done within its confines. The Access Review Committee assesses access requests by each company or researcher. Access to the data can only be for health research purposes, limited to the data they need in accordance with their application and research protocol, only allowed through a secure login, and as mentioned above, without downloading any data. Members of the GENE have to pay a fee for becoming members and having access to the GE data services. The fee for large companies (i.e., USD $1 billion market capitalization) is USD $320,000. The fee for smaller companies is USD $32,000. These fees cover part of the costs of storage, security, and analytic services. They also commit to having a specific number of employees devoted to the activities relevant to the project. GENE members can have a controlled access to up to 5,000 genomes and corresponding health information (78, 80, 89–91).

An independent Ethics Advisory Committee advises the GE board on participants’ consent, privacy, and confidentiality and on how to best ensure data’s security. GE will take all the measures necessary to maintain the security of the data. Some of these measures include removing identifying information from the raw data and encryption tools. The GE data center complies with the BS7799 and the ISO27001 security standards. Because the project is focused on England’s (and extending to Ireland’s, Scotland’s, and Wales’) population and the data remain in the GE’s data center, the project only commits to comply with U.K. legislation (e.g., Data Protection Act of 1998 and Access to Health Records Act of 1990) (77, 80, 89, 91).

The type of cloud provided by GE in this project is private, unlike the cloud in the MSSNG and the ICGC projects. The PPP differs in organization and in dynamics as well. Whereas in MSSNG and ICGC, the private sector was represented by the CCS providers, in the 100,000 GP, the CCSs are provided by the public sector in partnership with some private companies helping with the DNA sequencing and interpretation of data. Similar to the services provided by Google and AWS, the CCSs are scalable and elastic, as members can and are even encouraged to nominate new rare diseases and tumor types to be included in the project (92).

The companies’ early involvement in the research and their collaboration with researchers may accelerate the translation of basic research into concrete clinical innovative products, but the impact of restrictions on data downloading remain to be seen. Patients may have access to a better, timely, and more accurate diagnosis and treatment, given that any relevant information resulting from the sequencing and analysis of the participants’ samples will be returned to their doctors. Furthermore, health-care staff is trained with new skills and practices (78, 80, 90).

The expressed intention of the parties involved in this project is to achieve a better understanding of the involvement of genomics in cancer, rare disorders, and infectious diseases and the development of new diagnostic tools and personalized treatment that advances the field and the industry and improves patients’ health. This is mentioned in the project’s documents, description, and policies. However, their access and use model remain contentious. The reason for involving members of the public and the private sector and for assigning them the respective roles they have is based on two recognitions: that the private sector has always had an important role in the development of innovative medical products and that an early involvement of all its parties may help to employ resources more efficiently and to accelerate the R&D process (77, 78, 86).

## Looking Forward: Lessons Learned

The three case studies presented have benefited from being PPPs. Google’s and AWS’ private cloud-computing infrastructure and services have considerably empowered the MSSNG and the ICGC research projects by enhancing the value of the genomic and health data they have collected in the following manner. First, the IT companies’ infrastructure and services enable a global and multidisciplinary network of researchers that can collaborate and share their ideas, knowledge, experience, skills, and resources. Collaboration and sharing enhances the value of that genomic and health data, as more R&D can result from that data. Second, the global aspect of Google’s and AWS’ services (e.g., the servers located in different regions) requires them to comply with laws of several jurisdictions. This increases the possibility of expanding the projects to larger geographical areas. Third, MSSNG and ICGC are able to use this infrastructure and these services without having to put up large capital investments for their setup and maintenance. This helps to democratize the innovation process, as it allows small- and medium-sized research centers and companies from developed and developing countries to get involved in such large research projects. Fourth, having Google and AWS as the companies responsible for providing CCS can add reliability and trust in the minds of the participants and the other partners of the projects. The reason for this potential increase in reliability and trust is that both companies have a long, well-established experience in handling big data. This experience may translate into having the necessary resources and skills to have strong security measures. Furthermore, having these companies not only as CCS providers but also as partners in these projects can result in more specially tailored and better suited terms of service, as the companies have an invested interest in the success of the projects. When customers simply hire CCSs as opposed to forming a PPP with the companies that provide them, they usually have to adapt to the terms under which they generally provide their services. This is problematic given that most of these terms of use state limited responsibility for the integrity and mobility of the data. These limits are handled differently when the CCS providers have a larger interest invested in the project, as in the case of MSSNG and ICGC.

Some of the benefits found in these two cases are also observed in the 100,000 GP, including, above all, links to health-care data in a network of researchers and innovators who share their ideas, knowledge, experience, skills, and resources, thereby increasing the value of the genomic data and the potential of a more efficient R&D process. However, given that in this case, the CCSs are provided by a private in-house data center, there are certain differences. For instance, the 100,000 GP only complies with the laws of the U.K. This makes it difficult to expand the project to other regions. Second, this project had to make a considerable initial capital investment to set up the data center and the cloud-computing infrastructure. Third, contrary to MSSNG and ICGC, the 100,000 GP maintains exclusive control over the data. This could increase the trust that users and participants may have on the protection of their privacy but decrease access and use for research and downstream benefits for patients. Moreover, given the limited experience that GE has in comparison with Google or AWS in handling massive amounts of data, some participants may in fact doubt GE’s capacity to effectively protect the donors’ data. Yet, the participation of the private sector in the sequencing and translational process in this project is expected to accelerate the R&D process, as they bring in their infrastructure and their scientific, technological, and budgetary experience into the process. Furthermore, having such an early involvement of private companies in the research process allows all parties to share their expectations and to contribute their opinions and their interests (e.g., nominating new rare diseases) to the general coordination and management of the partnerships challenges.

Overall, it could be said that while being organized as PPPs and employing CCSs, the three projects have advanced quickly and are organizing themselves to be important sources of future personalized medicine. Nevertheless, the benefits and achievements that the three projects have had, there still are unresolved matters. The first is the protection of confidentiality and privacy. The three projects implement, to the best of their abilities, security measures (e.g., encryption, removal of identifying information, controlled access, privacy and data sharing policies, etc.) that aim to maintain the confidentiality and privacy of the participants. The 100,000 GP even provides that the data will never leave their data center, which may undermine further research and innovation. In all the three, however, the re-identification of the participants remains a possibility due to the necessary linkage with health records. The second concern is the differences in expectations and goals that the different partners may have while joining and participating in a project. In the MSSNG and the ICGC projects, the stated reasons for Google and AWS to become partners do not seem to contradict the incentives of the partners of the public sector, namely, to advance the understanding of genetic diseases and to improve patients’ health with better diagnostics and personalized treatments. However, there may be other untold goals. For example, advertising is the source of the majority of Google’s revenues (93). Furthermore, Google is deeply involved in the development of artificial intelligence (94, 95). Given Google’s other possible interests, it is important that projects develop robust and transparent governance structures and conflict of interest policies to secure public trust. On the other hand, in the 100,000 GP, its private partners have a strong economic expectation/incentive that may run contrary to the scientific/medical goals. It should be stated, however, that this may also occur later on in the MSSNG and ICGC projects when the projects enter the translational phase. The third issue is the possible loss of control of the data, particularly in the case of the MSSNG and the ICGC projects that allow downloading of data for more diverse and enriched analysis. While the data are stored and analyzed in data centers with strong security measures and while they grant MSSNG and ICGC control over their data, neither of the leading institutions in these projects is in charge of the management, implementation, and decision-making regarding the security measures. Finally, as regards the 100,000 GP and its private data center, the fact that the data never leave the data center causes underutilization of the project’s data. This characteristic may also be perceived as in conflict with some of the common principles of open science (e.g., open data sharing, fast dissemination of knowledge, cumulative R&D, etc.) (96), as any data sharing will be very limited.

An important issue in our analysis of all the three cases is that we could not confirm whether some of the suggestions to overcome challenges found in PPPs that we mentioned earlier are actually being implemented, as our sources of information were mute in this respect.

## Conclusion

Public–private partnership is a useful and effective collaborative R&D model. It is particularly relevant for projects where the R&D process cannot be fully taken on by the public or the private sector alone. PPPs enable members of both sectors to come together and increase the availability of multiple resources and diverse knowledge and expertise that can make the innovation process less burdensome and costly, more efficient, effective, shorter, and with a wider reach. It also makes the R&D process more efficient, as it allows objectives and needs of the parties involved in the different stages of the R&D process to be known and addressed. In this respect, particularly in genomic research, the model of PPP facilitates the translation of basic to clinical research and it enables the use of CCSs.

Cloud-computing services increase the usefulness of genomic data for understanding disorders with a genetic component and developing improved and more personalized diagnostic tools, drugs, and treatments. They achieve this by enlarging the data users’ network in two ways. They can extend the geographical availability of the genomic data they store. They make it easier and cheaper for all users to access, analyze, and store their own and others’ data, regardless of whether they are or belong to small- and medium-sized centers from developed or developing countries. As a result, there has been an increase in the use of CCSs in health-care services and health-care research.

The three projects we discussed in this article benefit from the PPP model as well as from the use of CCSs, despite the differences in the way they are organized. However, there are two important matters that should be kept in mind. First, protection of data and privacy is essential for cloud computing to fully benefit and spur medical and genomic research. End-users are likely to have more trust in CCSs when they perceive that their data and privacy are properly protected. The more trust end-users have in the cloud, the bigger the network of users and projects using the data in the cloud will be. The larger the network of contributors, the faster the progression in genomic and medical research could happen and the more chances that patients will have to benefit from personalized healthcare (22). Second, even though the benefits of PPPs are palpable, this collaborative model still presents certain challenges. Large-scale genomic projects can be of long duration and cover a sizable geographical area. They can include a variety of partners and contributors with different and potentially conflicting objectives, resources, expectations, and viewpoints. Early integration of all parties, timely and appropriate distribution of functions and responsibilities, frequent monitoring of results, constant communication and trust among the partners, and adequate governance frameworks are helpful in managing those complications and increasing the chances of a successful PPP, provided also that the public is kept involved and informed about the project (3, 4).

Learning from the experience of past PPPs is critical in order to avoid making the same mistakes in the future. Therefore, research on those past PPPs to document the projects’ achievements and failures as well as to provide information on the development and outputs of such partnerships would provide helpful guidance for future PPPs (3, 7). In addition, we propose that guidelines on best practices to adequately manage personal information stored in clouds and transferred online for research purposes be established to foster the protection of data integrity and privacy. These guidelines should enable research institutions to maintain control over the use of the data during and after research projects conclude and encourage data transfer in accordance with open science principles.

## Author Contributions

PG drafted the article. PG, YJ, and BK contributed to the design of the article, interpretation of the data, critical revision of the paper contributing to its intellectual content, and approved the final version. They all agreed to be accountable for all aspects of the article.

## Conflict of Interest Statement

The authors declare that the research was conducted in the absence of any commercial or financial relationships that could be construed as a potential conflict of interest.

## Footnotes

^1^Big data can be defined as vast amounts of data with significant variety or heterogeneity that can be accessed and analyzed to reveal patterns, trends, and correlations [Dimitrov (10), p. 159; Costa (11), p. 433; Jordan (12), p. 5].

^2^The IaaS provides storage, networks, and analytical/processing computing services for application program interfaces (APIs) to migrate workloads and data to a virtual machine. In this case, users have a determined storage capacity, processing capability, and other resources that they can use to start, stop, access, and configure the virtual machine they have been assigned. They are allowed to install their own operating systems and applications on the infrastructure provided [Mell and Grace (20), p. 3; Dove et al. (15), p. 1272; Kuo (22), p. 2; Marston et al. (21), p. 178].

^3^The PaaS hosts development tools that are made available *via* APIs, browsers, or software to develop and deploy software applications onto a cloud infrastructure [Mell and Grace (20), p. 2 and 3; Dove et al. (15), p. 1272; Kuo (22), p. 2; Marston et al. (21), p. 178].

^4^The SaaS allows users to deliver software applications and run them on a cloud infrastructure. The applications are accessible from the users’ devices through the use of a web browser or a program interface, without the need to install the application in a particular computer [Mell and Grace (20), p. 2; Dove et al. (15), p. 1273; Kuo (22), p. 2; Marston et al. (21), p. 178].

^5^The MBaaS provides a way to link web applications and mobile applications to a cloud infrastructure.

^6^An example of anonymity tools is the use of a biological PIN code that allows a donor to provide a sample without any identity data. In this case, the registration of the sample includes only the individual’s unique biological characteristics without enabling any link back to the donor [Dove et al. (15), p. 1271].

^7^Cloud-computing services in telecommunication/teleconsultation allow actors in the area of healthcare (e.g., medical practitioners, specialists, clinicians, nurses, paramedics, etc.) to communicate and share data. An example of this case would be collecting, accessing, sharing, and analyzing the data of a particular patient from different hospitals or health-care providers (e.g., patient records and medical history). For instance, the Cloud Cardiology enables medical professionals to share ECGs simultaneously with members of the hospital and with other practitioners outside the hospital [Griebel et al. (16), p. 4].

^8^Cloud-computing services are used in the area of medical imaging to store, share, and process images. An example of this type of images would be X-rays. For instance, Accenture Medical Imaging Solution is built to review X-rays, MRI, and CT scans [Griebel et al. (16), p. 7; Ahuja et al. (24), p. 15].

^9^Cloud-computing services enable broad access to health data concerned with disease prevention, health promotion, and improvement of the population’s health. For instance, they may be used to store personal health and lifestyle data collected by mobile devices and extract patterns that would help prevent epidemics and improve the population’s health. They can also be used to monitor the progress of conditions such as post-traumatic disorder syndrome (Tele-PTSD Monitor) or to provide specialized training routines after receiving physiological data (iFit) [Griebel et al. (16), p. 8].

^10^Cloud-computing services allow hospitals to digitalize health records, update clinical processes, and give access to medical staff at anytime and from anywhere. They can also provide faster and more accurate billing [Kuo (22), p. 4].

^11^Cloud-computing services help to host and operate applications for planning, managing, and assessing therapeutic interventions. For instance, VirtuaLinac is a web application used to model radiation treatment components. iSMART is a cloud-computing web server that provides access to information on over 20,000 compounds of traditional Chinese medicine that focuses on identifying compatible and derivative compounds that could be useful for drug research [Griebel et al. (16), p. 9; Chang et al. (25)].

^12^The U.S. Cloud First policy was launched in February 2011. Its purpose was to encourage the governmental agencies to evaluate the possibility of implementing safe and secure cloud-computing options before making any investments in IT infrastructure. With this policy, the U.S. government was aiming to be more efficient, agile, and innovative (26).

^13^The term “covered entities” refers to any health plans, health-care clearinghouses, and any health-care provider who transmits health information in electronic form in connection with transactions for which the Secretary of HHS has adopted standard under HIPAA [US Department of Health and Human Services (28), p. 2].

^14^Individually identifiable health information refers to “information that relates to the individual’s past, present, or future physical or mental health or condition, the provision of health care to the individual or the past, present or future payment for the provision of healthcare to the individual, and that identifies the individual or for which there is a reasonable basis to believe can be used to identify the individual.” [US Department of Health and Human Services (28), p. 4].

^15^Business associate is defined as a person or organization, different from a member of a covered entity’s workforce that performs certain functions or activities or that provides certain services to a covered entity involving the use or disclosure of individually identifiable health information. Some of these activities include claims processing, data analysis, utilization review, and billing. The services are legal, actuarial, accounting, consulting, data aggregation, management, administrative, accreditation, or financial [US Department of Health and Human Services (28), p. 3].

^16^Some of the rules stated by HIPAA to protect health information are the following. (1) That covered entities must have policies and procedures to maintain their workforce’s access and use of protected health information to the minimum amount reasonably necessary and in accordance with their specific roles. (2) That they must also have privacy practices. (3) That companies and organizations are prohibited from sharing personal health data to non-affiliated parties. (4) That the use or disclosure of an individual’s protected health information by a covered entity requires the written consent of such an individual or that such use or disclosure falls within the allowed or required uses or disclosures stated by the privacy rule. Required disclosures are those that have to be made to the individuals when they request access to their information and to HHS when an investigation, review, or enforcement action orders it. The allowed uses and disclosures are those made to the individual, those in association with treatment, payment and healthcare operations, those that serve as an opportunity to agree or object, as an incident, those based on public interest and benefit activities, and those made regarding limited data for purposes of research, public health, or health-care operation. (5) That the use or disclosure of limited data set (protected health information from which specific direct identifiers have been removed) for purposes of research, public health (i.e. to avoid a serious threat to health or safety) or health-care operation is among the permitted uses or disclosures without the need of the individual’s consent. (6) That genomic data stripped of identifiers may not be considered protected health information, and therefore, the use or disclosure of de-identified health information is not restricted [US Department of Health and Human Services (28), p. 4, 9, and 20].

^17^Name, age, ethnic origin, and medical records are just few of the forms of information considered personal.

^18^For more information on the first two examples, see for (1) Collaborative Cancer Cloud: Dana-Farber Cancer Institute and Ontario Institute for Cancer Research Join Collaborative Cancer Cloud | Ontario Institute for Cancer Research,” *Ontario Institute for Cancer Research*, March 31, 2016, http://oicr.on.ca/news/news-releases/dana-farber-cancer-institute-and-ontario-institute-cancer-research-join-collaborative-cancer-cloud; “Dana-Farber Cancer Institute and Ontario Institute for Cancer Research Join Collaborative Cancer Clo,” *Oregon Health & Science University*, March 31, 2016, https://news.ohsu.edu/2016/03/31/dana-farber-cancer-institute-and-ontario-institute-for-cancer-research-join-collaborative-cancer-cloud and for (2) Cancer Genome Collaboratory: The Cancer Genome Collaboratory | Genome Canada,” *Genome Canada*, accessed September 7, 2016, http://www.genomecanada.ca/en/cancer-genome-collaboratory and “Main—Bionimbus Protected Data Cloud,” accessed September 7, 2016, https://bionimbus-pdc.opensciencedatacloud.org/.

^19^This is study is found as Yuen et al., “Genome-wide characteristics of the novo mutations in autism” (37), *npj Genomic Medicine*.

^20^Research data include information concerning the participants such as family configuration, age at time of testing, sex psychopathology, diagnosis, cognitive functioning, family and medical history, and other relevant clinical information, without including any personal identifying information.

^21^A persistent disk can be attached to different virtual machines retaining the data; so if a virtual machine is terminated, the disk can be attached to a new one. On the other hand, local SSD is physically attached to the server hosting the virtual machine.

^22^The NHS includes 27 institutes all of which are part of the U.S. Department of Health and Human Services [NIH Press (55)].
